# Gender Differences in Meaningful Leisure Among Older Adults: Joint Displays of Four Phenomena

**DOI:** 10.3389/fpsyg.2018.01450

**Published:** 2018-09-07

**Authors:** Nuria Jaumot-Pascual, María Jesús Monteagudo, Douglas A. Kleiber, Jaime Cuenca

**Affiliations:** ^1^Department of Counseling and Human Development Services, University of Georgia, Athens, Georgia; ^2^Faculty of Social and Human Sciences, University of Deusto, Bilbao, Spain

**Keywords:** aging, gender, meaningful leisure, mixed methods, joint displays

## Abstract

Vital events, such as widowhood and retirement, are broadly accepted as points of inflection in the lives of older adults that often differ according to gender. In this study, we analyzed the influence of gender on meaningful leisure among older adults through the integration of qualitative and quantitative findings. The use of joint displays revealed that in this sample of people from Northern Spain: (1) there were no significant differences in the influence of retirement and widowhood on the leisure of the two genders, (2) the ethic of care was a constraining factor in older women’s leisure, (3) women were more innovative in their leisure in older age, and (4) volunteer activities were highly segregated by gender. The use of joint displays helped illuminate these four phenomena in light of quantitative and qualitative data.

## Gender and Leisure in Later Life

Life events, such as widowhood and retirement, are widely recognized as moments of vital transition for older adults (Antonovsky and Sagy, 1990). These events often set into motion personal innovation in later life (Kleiber and Nimrod, 2008; Nimrod et al., 2012). However, the changes older adults experience are generally thought to differ according to gender. In the case of women, whether and how women have been employed outside of the household and at what moment in life they become widows, both influence how and when developmental tasks are triggered (Lopata, 1973; Henderson and Allen, 1991; Cusack, 1994; Hurd, 1999; Lee and Bakk, 2001; Gibson et al., 2002, 2003). In addition, the ethic of care is a key constraining factor that limits women’s leisure at any age (Henderson and Allen, 1991). In the case of men, their leisure trajectories in older age often include continuity with previous pursuits in life and are colored by different conditions after widowhood than those of women (Carr et al., 2005; Genoe and Singleton, 2006; Van den Hoonaard, 2010).

### Developmental Tasks

The literature has characterized retirement and widowhood as moments of transition in human development when individuals question their life priorities due to the disappearance of previous obligations that occupied their time. Traditionally, retirement had this role for men (Antonovsky and Sagy, 1990) and widowhood for women (Lopata, 1973; Hurd, 1999; Lee and Bakk, 2001). Hurd (1999) pointed out that widows were especially likely to address developmental tasks after their loss if they had not worked outside the home or did gender-specific jobs (i.e., sewing/tailoring) that traditionally did not require contractual relationships (i.e., self-employment and piecework), and/or stayed married until older children no longer required their direct attention, i.e., factors that had been constraining to self-expression. As Lee and Bakk (2001, p. 54) asserted, once women became widows, “they develop their own sense of personal identity and begin to live full lives.” Lopata (1973) called this phenomenon “blossoming.” Lee and Bakk (2001) pointed out that after a period of adaptation, many women were able to continue their development and have meaningful lives and even relish their newfound freedom. Lopata’s (1973) own research revealed that many widows actually reported ‘compensations’ that follow the loss of a spouse such as living alone, a decrease in domestic responsibilities, and independence. The literature has shown that these transitions influence women’s leisure practices by liberating them from responsibilities that limited their leisure involvement (Nimrod, 2007; Nimrod and Kleiber, 2007; Campbell and Yang, 2011; Liechty et al., 2012). However, with the progressive incorporation of women into the workforce and changing gender roles, retirement and widowhood may also be changing in their function as developmental tasks, possibly making previous literature historically limited.

Since Baltes and Carstensen (1996) defined the process of successful aging, as essentially doing the best with what one has, the literature has shown the importance of leisure in contributing to the maintenance of a sense of well-being. Through the years, the literature has shown the links between leisure and successful aging in general (e.g., Paggi et al., 2016), and in adverse life situations, such as adapting to chronic illness (Kleiber et al., 2011; Hutchinson and Nimrod, 2012), and coping with traumatic injury and illness (Hutchinson et al., 2003). The literature has also studied these links with different populations, such as women of different racial backgrounds (Riddick and Stewart, 1994), and the LGBT older adult community (Fredriksen-Goldsen et al., 2014), and in different types of leisure, including serious leisure (Brown et al., 2008).

Mannell (2007) conducted a literature review of the evidence available on the impact of leisure on health and well-being and found that “Leisure, viewed as discretionary behavior outside of work and other obligations, positively influences physical, psychological and spiritual health and well-being through opportunities for making meaningful choices and the benefits provided by specific experiences” (p. 124). The review also associated leisure activity with identity formation and affirmation, effective coping in situations of stress and in adverse events, and positive influences on other domains of life such as work, family, and personal relationships. Such contributions make the study of leisure’s role in older adult development an important piece of the puzzle.

### Innovation in Leisure

Nimrod and Kleiber (2007) defined leisure innovation as an older adults’ engagement in new leisure activities after retirement or widowhood. They defined two types of innovation: self-preservation and self-reinvention innovation. In self-preservation innovation, “innovation represents an opportunity for renewal, refreshment and growth that is continuous in some respects from earlier interests and capacities” (p. 17). Self-reinvention innovation represents an opportunity to reinvent the self. Though innovation has been studied from a gender perspective (Campbell and Yang, 2011; Liechty et al., 2012; Jaumot-Pascual et al., 2016), it has always been studied using qualitative methodologies. In an interview study with 10 women and 10 men, Jaumot-Pascual et al. (2016) found that the older women in the study tended to be more innovative than men in their leisure. In this study, we use quantitative and qualitative methods to examine and further our understanding of leisure innovation as influenced by gender, the being the first mixed methods approach to the role of gender in leisure innovation.

After widowhood and retirement, the question turns to the content of the leisure that opens up before older adults. There is a considerable body of work addressing continuity (Atchley, 1999; Kelly, 1999), substitution (Rubinstein and Kilbride, 1992; Lefrancois et al., 1997), and decline and abandonment (Iso-Ahola et al., 1994) of activities in retirees’ involvement in leisure. However, there is not as much literature addressing innovation in later life (Nimrod and Kleiber, 2007; Liechty and Yarnal, 2009; Nimrod and Rotem, 2012). In a study of recently retired and well-educated elders in the southeastern United States, Nimrod and Kleiber (2007) found that participants in the study embraced new activities that bore relatively little resemblance to anything they had done before. Continuity was reflected to some extent, but even with activities that had some earlier form in a person’s life, the changing circumstances of the activity provided entirely new sources of meaning. Another recent study on innovation with retirement-age women (Liechty and Yarnal, 2009) established that innovation fosters further innovation. They confirm the positive experience of adding a new activity reinforces the participant’s ability and desire to add more activities.

### Ethic of Care

The ethic of care is a model of moral development developed by Carol Gilligan (1977) where the highest moral imperative requires taking care of others’ needs and sustaining relationships. Henderson and Allen (1991), following the work by Gilligan (1977, p. 97), provided a review and an interpretive framework for examining an ‘ethic of care’ “as it offers possibilities and constraints for the leisure of women.” They defined ‘ethic of care’ as:

Seeing and responding to need, of taking care of the world by sustaining the web of connection so that no one is left alone. […] Most females follow a developmental path that concentrates on responsibility and commitment to others and that women define themselves in relation to others (Henderson and Allen, 1991, p. 99).

Henderson and Allen (1991, p. 102) argued that an ethic of care shapes personal capacities and socialization factors “that define what opportunities may exist for leisure.” The ethic of care becomes internalized, impacting decisions on day-to-day basis, becoming a constraint when the needs of others come first and one’s own needs are put last. Because of the relational nature of women’s development (Belenky et al., 1986), “Meaning is not in an activity but in the context of the relationship and responsibility. Thus, the social benefit of leisure may be embodied in this ethic of care” (Henderson and Allen, 1991, p. 105), which means that women tend to choose activities where social interaction is either the main activity or a requirement for the activity to take place.

With the social changes currently occurring where gender roles are increasingly less distinct, we might expect for gender differences in the influence of the ethic of care on women and men to diminish. Studies such as Mailey et al. (2014), where both working mothers and fathers express having very similar constraints to physical activity, including caring for children, seem to indicate that this is the case. However, much of the literature continues to show that an ethic of care is a constraint for women’s leisure that limits their ability to participate in leisure activities (Miller and Brown, 2005; Skowron et al., 2008; Quinn, 2010; Offer, 2016). Joseph et al. (2015) showed that the impact of the ethic of care may be accentuated in certain racial and ethnic groups, such as the African American community in the United States. In a study of active aging and leisure in Spain, Lazcano et al. (2010) found that the ethic of care continued having a differentiated role according to gender, with family responsibilities being more of a barrier to women’s leisure than men’s. Also in Spain, Aristegui et al. (2015) found that being a caregiver was a constraint for 4.1% of participants. Though they did not disaggregate by gender, they found a slight tendency for those living in rural areas to be more constrained by caregiving roles.

Today’s older generation is a cohort that grew up and lived during a time when gender roles were more differentiated than currently; thus, one would expect research on the influence of the ethic of care on leisure to reflect this. However, we have not found a body of literature that explores the role of an ethic of care on *older* women’s leisure. Brown et al. (2009) found that, middle aged (45–50) women’s engagement in physical activity diminished after the birth of a grandchild. If we assume that this decrease is due to increased caregiving responsibilities, it would indicate that an ethic of care influences the engagement in physical activity among grandmothers. However, similar data and findings are absent for older women in the study, indicating that the role of an ethic of care in older women’s leisure is mostly unexplored. In this study, we contribute a preliminary understanding of the influence of the ethic of care on older women.

### Volunteering as Leisure

Volunteering is intimately related to the ethic of care, which provides the motivation to get involved in volunteering. Henderson and Allen (1991) highlighted an ethic of care as an underlying value for volunteerism and community service.

The ethic of care embodied through the social reform movements of a number of groups of women has created many of the social institutions that we have today. […] The ethic of care helps to provide a basis for focusing on others through volunteerism and community service, which can be important leisure experiences for many people (Henderson and Allen, 1991, pp. 105–106).

In the context of Spain, Villar et al. (2013) conducted a study using data from a survey of older adults in Spain done by IMSERSO (the Institute for Older Adults and Social Services) in 2010 and found that “Gender […] stands out as relevant in three of the four generative activities [caring for grandchildren, caring for dependent adults, volunteering, and civic participation] that we have taken into account. In this sense, both the care of dependents and volunteering are activities (especially this last one) heavily feminized” (p. 73, citation translated by this article’s authors). Again, Villar et al. (2013)’s work suggests an intimate connection between volunteering and the ethic of care, this time within the Spain context.

Rotolo and Wilson (2007) found that, there were substantial differences in the content of the activities women and men do as volunteers, at least in the United States. They found that “men are more likely to occupy leadership positions than women. They are more likely to do maintenance work and teach or coach, while women are more likely to prepare and serve food or clothing, raise money, and ‘help out’ at events” (p. 559). In this same vein, Manning (2010, p. 128) found that, older women “are more likely to be involved in volunteer behavior characterized as more caring, person-to-person tasks, compared to men, who are more likely to be involved in political or public leadership positions,” indicating that the ethic of care influences the type of volunteer women and men choose to get involved in.

## Methodology

This paper addresses a purely mixed methods question: how do the quantitative data and the qualitative findings provide further understandings around gender differences among older people in the Basque Country, in Northern Spain? We break down this umbrella question into four, phenomenon-specific questions in the findings section.

### Mixed Methods

In our research, we wanted to avoid taking an a-paradigmatic stance by simply using “what works” (Greene, 2007; Hall, 2013), and adopted Morgan’s (2007, p. 71) definition pragmatism, according to which “The pragmatic approach is to rely on a version of *abductive* reasoning that moves back and forth between induction and deduction—first converting observations into theories and then assessing those theories through action” and where the “process of inquiry … evaluates the results of prior inductions through their ability to predict the workability of future lines of behavior” (p. 71). In this study, this abductive reasoning in the secondary analysis of the data led us to evaluate the results of the analysis of the interviews in light of the results from the quantitative data.

This study was conceived as an explanatory, sequential, mixed methods design (Creswell, 2015, p. 60) where the results of the qualitative method were to be used to explain the quantitative findings. The resulting qualitative and quantitative data were, initially analyzed independently. In this paper, we mix methods by analyzing the data in an integrated manner through joint displays (Onwuegbuzie and Dickinson, 2008; Fetters et al., 2013) to see how the quantitative data and the qualitative findings provide further insights around gender differences in meaningful leisure activities among older people in the Basque Country. This study answers the following four mixed methods questions: (1) Does widowhood play the role of retirement in older women’s development? (2) Are women more innovative in their leisure than men after older adulthood transitions? (3) Does an ethic of care constrain older women’s leisure involvement? and (4) What differences are there among older women and men in volunteer participation?

### Study Design, Instruments and Sampling Strategy and Ethical Issues

#### Study Design

This secondary analysis falls under the umbrella of a larger research project funded by the regional government of Spain’s Basque Country to study successful aging and meaningful leisure among older adults through a mixed methods study. The larger research project was a mixed methods study that utilized two main data collection methods: a questionnaire and a semi-structured interview. The study’s design was conceived as an explanatory, sequential, mixed methods design (Creswell, 2015, p. 60) where interview volunteers were drawn among questionnaire respondents and the results of the qualitative method were initially used to explain the quantitative findings in the first article (Cuenca et al., 2014). In his article, the team conducted quantitative analysis of the data as a whole using non-parametric chi-square analysis, Pearson correlations between variables and regression analysis, and preliminary thematic qualitative analysis that focused on theoretical constructs such as serious leisure and well-being. The qualitative data were analyzed independently from the quantitative data and reported in a second article (Jaumot-Pascual et al., 2016). The team used qualitative narrative analysis (Polkinghorne, 1995) from a gender perspective that highlighted the complexity of gender differences in the construction of meaningful leisure.

In light of these qualitative findings, we decided to conduct further analysis that brought together quantitative and qualitative data from a gender perspective. Toward this end, we conducted secondary data analysis for this manuscript, where we integrated the two sets of data through joint displays (Onwuegbuzie and Dickinson, 2008; Fetters et al., 2013). In the construction of joint displays, we paired qualitative findings with questionnaire items addressing the same topics for further insights that were not accessible through one set of data, such as complementing our qualitative finding that the types of volunteering activities were highly segregated by gender with whether there were differences in volunteering rates by gender.

#### Instruments and Sampling Strategy

The questionnaire sampling method was stratified according to gender, age, and place of residence (i.e., urban, rural) in the three provinces of the Basque Country to get a representative sample of older adults. Participants were approached, by researchers in public settings, such as malls, libraries, senior centers, and town squares, in urban and rural locations. A total of 755 questionnaires were completed. The participants were approximately equally divided by gender, and the sample was similarly distributed in three 5-year age ranges that spanned from 61 to 75 years old (see **Table 1**). The questionnaire included mostly closed-ended questions about sociodemographic information, leisure involvement, and subjective well-being.

**Table 1:** Sociodemographic characteristics of questionnaire respondents.

Gender	Age	Place of residence
362 women 48%	239 aged 61–65 years 31.7%	143 rural^∗^ 18,9%
393 men 52%	266 aged 66–70 years 35.2%	343 urban^∗∗^ 45,4%
	250 aged 71–75 years 33.1%	269 capital city^∗∗∗^ 35,6%

*^∗^Rural: fewer than 10,000 inhabitants. ^∗∗^Urban: 10,000 inhabitants or more. ^∗∗∗^Capital city: inhabitants of capital cities*.

The goal of the interviews was to study the paths of leisure involvement of older adults who showed patterns of meaningful leisure involvement. The interviewees were selected using criterion sampling based on their answers to the questionnaire. The criteria for the selection of interview participants were: (1) being 61–75 years old, (2) living in the Basque Country, (3) being involved in at least one leisure activity that they regarded as especially meaningful, (4) having expressed high levels of satisfaction with their especially meaningful leisure activity, and (5) having expressed their willingness to be interviewed. Out of the 375 respondents who agreed to be interviewed, 37 fulfilled all the criteria. Further narrowing down was done according to gender and locale of residence to ensure roughly equal representation for the two. This resulted in the selection of twenty interviewees—10 men and 10 women (see **Table 2**). Members of the research team conducted face-to-face, approximately 1 h, recorded interviews over the course of 6 months at the local university, which were subsequently transcribed verbatim.

**Table 2:** Sociodemographic characteristics of interviewees and meaningful leisure activities.

Gender	Age	Place of residence	Meaningful leisure activities
10 women	62–75 years old	1 rural^∗^ 1 urban^∗∗^ 8 capital city^∗∗^	3 Taking courses (photography, languages, and culture) 2 Physical activity 2 Traveling 1 Writing poetry 1 Caring for grandchildren 1 Volunteering
10 men	61–68 years old	1 rural^∗^ 4 urban^∗∗^ 5 capitalcity^∗∗^	2 Cooking 2 Singing and teaching in a choir 1 Painting 1 Traveling 1 Taking courses (language) 1 Hiking 1 Playing cards 1 Photography

*^∗^Rural: fewer than 10,000 inhabitants. ^∗∗^Urban: 10,000 inhabitants or more. ^∗∗∗^Capital city: inhabitants of capital cities*.

#### Ethical Issues

The University of Deusto Research Ethics Committee provided ethical approval for the study and a written informed consent was obtained from participants. Aspects such as the recruitment of the people of the sample, applied informed consent procedures of participation, confidential and anonymous management of personal data and interaction with people were all suitably addressed from an ethical standpoint.

### Data Analysis

As we stated in the study design section, the team has previously analyzed the study’s quantitative and qualitative data independently, resulting in two published articles (Cuenca et al., 2014; Jaumot-Pascual et al., 2016). In the current secondary analysis, we bring together the quantitative and qualitative data from a gender perspective. The existence of quantitative data that were directly related to the qualitative findings permitted integrating the findings from the two sets of data through mixed methods joint displays that provided insights that go beyond the findings for each type of data. In this equal-status mixed research, the quantitative analysis is variable-oriented and the qualitative analysis is case-oriented (Onwuegbuzie et al., 2009). We employed the findings generated in the case-oriented qualitative analysis as the organizational framework on which to integrate the variable-oriented quantitative analysis. This integration occurred at the interpretive and reporting level through a joint display, once data had already been analyzed.

Using Onwuegbuzie and Dickinson (2008)’s taxonomy of visual representation, the joint displays we have created to represent the integrated themes take the shape of a thematic comparative, level 3 text-table, where theoretical constructs are matched with both qualitative and quantitative findings from the study to make the points of connection and the extensions of the findings apparent. The team analyzed the quantitative questionnaire item responses using non-parametric chi-square analysis and matched them with the qualitative themes developed in the previous Jaumot-Pascual et al. (2016) analysis.

### Validity

We ensure the quality and reliability of our meta-inferences generated through the integration of findings by using Tashakkori and Teddlie (2008)’s integrative framework. Quality and rigor have been the norm in the study for its duration, from its design to the interpretation stage. From the criteria described by Tashakori and Teddlie, we highlight the careful work of interpretive distinctiveness, where team researchers have developed nuanced conclusions that bring a new perspective to broadly accepted constructs in leisure studies.

### Limitations

One of the limitations of this study stems from the fact that the qualitative data collection was designed to study interviewees’ leisure itineraries, trying to reconstruct their vital trajectories as they related to leisure. Focusing on this group’s meaningful leisure, we looked, at the moment, in the vital cycle where the preference or interest for a meaningful activity emerges, which is the moment when the leisure activity starts from the perspective of leisure innovation. Although the quantitative data collection also included some items on leisure itineraries, these have not been used in the joint displays analysis. In summary, for this secondary analysis, data have been analyzed in ways that were not foreseen in the design phase of the study, which may have inserted unforeseeable limitations.

As in any study, the analysis of the data could have taken different directions, which means that other findings could have been possible. In this case, we focused on the analysis of questionnaire items that were directly related to our qualitative findings. This meant that covariates such as socioeconomic status, objective health and physical limitations, and social networks, among others, were not taken into account in our analysis because they did not align with our goal for the current study of looking at our four phenomena of interest from a gender perspective. There are also other factors that might influence the phenomena of leisure innovation and the influence of the ethic of care on women’s leisure, such as satisfaction with leisure. These are questions to be addressed in the future.

## Findings

We use our main theoretical constructs to organize the contrast of qualitative and quantitative findings and to present our meta-inferences. In this manuscript, we tackle four main theoretical constructs: widowhood and retirement as developmental tasks, women as more innovative in their leisure after widowhood and/or retirement, the ethic of care as a constraint to women’s leisure, and volunteering as a gender segregated activity. Further discussion of the meta-inferences can be found in the discussion and conclusions section of the article.

### Widowhood and Retirement as Developmental Tasks

In the analysis of the qualitative data, we found that most women indicated that retirement and, especially, widowhood made them reconsider their lives and motivated changes in their leisure practices. Retirement played this role for men, but to a lesser extent, mostly by allowing them more time to practice lifelong leisure activities.

In the questionnaire, we analyzed the answers to the question “Can you tell us whether during the last years anything has happened that has meant a change in your leisure practices?” The answer options were yes or no, which were then followed by two open ended questions about which changes and when they happened. We found that women had to change their leisure practices due to events in their lives significantly more often than men (χ^2^ = 11.315, *p* = 0.001). Retirement influenced both women and men’s leisure at similar rates (12.2% of men, *std. residual* 0.7; 9.7% of women, *std. residual*−0.8). The results suggest that widowhood influenced leisure approximately as often for women as for men (3.1% of men, *std. residual* −1.8; 7.5% of women, *std. residual* 1.9), with a difference that was not significant. Thus, the data show that both widowhood and retirement influenced leisure changes among the older women and men on this study to a similar extent (see **Table 3**).

**Table 3:** Developmental tasks by gender.

Phenomenon: Retirement is a developmental task that creates discontinuities in behavior, in this case in leisure behaviors (Antonovsky and Sagy, 1990). For women who have been homemakers, widowhood plays the role of retirement in their development (Lopata, 1973; Lee and Bakk, 2001).		

**Qualitative findings**	**Quantitative findinss**	**Meta-inferences**
Women indicated that retirement and especially widowhood made them reconsider their lives and motivated changes in their leisure practices. Retirement played this role for men, but to a lesser extent. “My life has changed since I became a widow. It’s been a radical change. It was 11 years ago and I’ve had to get used to a different lifestyle. Afterwards I started traveling with friends and doing things” (Paz, woman).	Retirement impacted both Women’s and men’s leisure at similar rates (12.2% of men, std. residual 0.7: 9.7% of women, std. residual -0.8). Widowhood impacted leisure almost twice as often for women as for men (3.1% of men, std. residual -l.8; 7.5% of women, std. residual 1.9), though the difference was not statistically significant.	The quantitative findings diverge from both the literature and the qualitative findings. We speculate that the quantitative findings reflect the changing gender roles in Western societies where there are fewer differences in the roles the genders play inside and outside of the home.

### Older Women, Leisure Innovation, and Later Life Transitions

Our analysis addressed the notion that older women are more innovative in leisure, meaning that they start new leisure activities after later life transitions more often than men from the perspective of the Leisure Innovation Theory of Successful Aging (Nimrod and Kleiber, 2007; Nimrod and Hutchinson, 2010; Nimrod and Rotem, 2012) and the findings by Iso-Ahola et al. (1994). We operationalized the innovation variable through the beginning of new leisure activities after retirement or widowhood, understanding these beginnings as an expression of the openness to new interests.

In the analysis of the qualitative data we found that women tended to report that they started new leisure activities (meaningful or otherwise) after later life transitions, while men tended to continue the practice of lifelong leisure activities. This also manifested in the fact that, through their lifelong participation in these activities, they acquired skills that they were passing down to others in later life.

In the quantitative phase of the study, we considered the starting point for participation in leisure activities and their maintenance through time as indicators of commitment to their practice. In previous studies (Cheng et al., 2017; Ronkainen et al., 2017), it has been shown that practicing an activity for 10 or more years approaches what is understood as serious leisure. Thus, we selected the 10 years mark as an indicator of the longevity of a leisure activity. In the questionnaire, we analyzed the answers to the question “I practice [meaningful leisure activity] since…” Response options included less than a year, between 1 and 3 years, between 4 and 6 years, between 7 and 10 years, and more than 10 years. We found that women had not participated in their meaningful leisure activity for as long a period of time as men (χ^2^ = 11.292, *p* = 0.046). A little less than two thirds of women (63.7%) had been practicing their meaningful leisure activity for over 10 years, and almost three fourths of men (74.5%) had. In addition, the percentage for women was higher in all other categories that indicate that they had practiced their leisure activity for shorter periods of time (less than 1 year, 1–3 years, 4–6 years, 7–10 years) (see **Table 4**).

**Table 4:** Gender and leisure innovation.

Phenomenon: The literature seems to indicate that older women are more innovative in leisure, meaning that they start new leisure activities after retirement and/or widowhood more often than, men (Iso-Ahola et al., 1994: Nimrod and Kleiber, 2007).		

**Qualitative findings**	**Quantitative findings**	**Meta-inferences**
Women reported that they started new leisure activities (meaningful or otherwise) more often than men after widowhood and/or retirement. Men tended to continue the practice of lifelong leisure activities more than women. “I have always enjoyed poetry. I don’t know why. Even as a child … I could not [write] before becoming a widow because I was too busy … How do I get my inspiration for my poetry? I don’t even know” (Blanca, woman). “I have always gone to the mountains. What happens is that after retiring I have had more time: (Nicolas, man).	Women had not participated in their meaningful leisure activity for as long as man (Pearson Chi-Square = 11.292, *p*- value = 0.046). While 63.7% of the women had been participating in their meaningful leisure activity for over 10 years, 74.5% of the men had.	The length of time women and men had participated in leisure activities is an indicator of leisure innovation in older age. Men’s activities had been part of their lives for longer, and they tended to continue them in older adulthood, while women’s activities were newer, indicating more leisure innovation in older age than men.

### Ethic of Care as a Constraint to Older Women’s Leisure

In the analysis of the qualitative data we found that most women reported that they cared for family members, mostly parents and grandchildren, and that this limited their participation in leisure activities. No men reported family responsibilities as a constraint for their leisure.

In the questionnaire, we analyzed responses to two questions: “At some point I have abandoned the practice of [meaningful leisure activity]” and “The main reason for my temporal abandonment of [meaningful leisure activity] was…” The response options for the first question were yes or no, and for the second one they were lack of time, health problems, lost of interest, family responsibilities, economic difficulties, lack of companion, found other more interesting activities, it was too difficult, it was too demanding, and other. We found that there was no significant difference as to how much women and men abandoned the practice of meaningful leisure activities (χ^2^ = 2.622, *p* = 0.270). However, our quantitative data illuminated the qualitative findings by showing that the reasons they abandoned the practice of meaningful leisure activities was significantly different by gender (χ^2^ = 14.271, *p* = 0.047). While only 4.5% of men indicated family responsibilities as the main reason for abandoning the activity, 24.4% of the women did; thus, over five times more women than men answered that events involving relationships with others influenced their leisure. The events that had an influence on women’s leisure were relational (32.1% of women, *std. residual* 2.3; 10.6% of men, *std. residual* −2.6). Within their relational nature, the events that influenced women’s leisure showed a much greater diversity, including family responsibilities (such as caring for grandchildren, parents, and partners), widowhood, children’s emancipation, separation or divorce, partner’s retirement, and family crisis. For men, on the other hand, these events were much more limited, including family responsibilities and widowhood exclusively (see **Table 5**).

**Table 5:** Ethic of care and leisure by gender.

Phenomenon: The ethic of care is a constraining factor on women’s leisure involvement by limiting their ability to participate in leisure activities (Henderson and Allen, 1991).		

**Qualitative findings**	**Quantitative findings**	**Meta-inferences**
No men reported family responsibilities as a constraint for their leisure, while 7 of the 10 women reported that they cared for family members, mostly parents and grandchildren and that this limited their participation in leisure activities. “I have my mother living with me. She is old, 94 years old, so I take care of her … [and] it does keep me from doing other things” (Silvia, woman).	Family responsibilities is the fourth reason cited for the temporary abandonment of leisure activities. The reasons for abandonment of leisure activities was significantly different by gender (Pearson Chi-Square = 14.271, *p*-value = 0.047). While only 4.5% of men indicated family responsibilities as the main reason, 24.4% of the women did. The events that had an impact on women’s Leisure were relational (32.1% of women, std. residual 2.3; 10.6% of men, std. residual -2.6). The decisions for the temporary abandonment of leisure activities due to family responsibilities are much more frequent among women: 13.6% of men that have temporarily abandoned a leisure activity as opposed to 86.4% of women.	Both the quantitative and the qualitative findings indicate that an ethic of care, as evidenced by the relational nature of the events that had an impact on participants’ leisure, had a larger role in the abandonment of meaningful leisure activities for women than for men.

### Participation in Volunteering Activities

In the analysis of the qualitative data, we found that many interviewees, both women and men, reported volunteering in their leisure time. The main difference between women and men in the qualitative data was in the content of the tasks they engaged in when they volunteered: women had mostly subordinate roles, such as helping with the organization of events and caring for people, and men had leadership and knowledge transfer roles, such as teaching the skills they had learned in their lifelong leisure pursuits.

In the questionnaire, we analyzed responses to the question: “Please indicate whether you participate in the following leisure activities. For those activities that you currently participate in, choose the answer option that better describes how it has changed due to the event indicated in [the previous] question.” Volunteering was one of 29 activities that participants addressed. The answer options were yes and no, and the yes was subdivided in four options: new activity, increased participation, continuation of participation, and decreased participation. In the analysis we found that there was a significant difference in the rate women and men volunteered. More than twice the amount of older women volunteered than men, with 15% of women volunteering as opposed to 6.2% of men (χ^2^ = 15.721, *p* = 0.000) (see **Table 6**).

**Table 6:** Participation in volunteering activities.

Phenomenon: Volunteering is a gender-segregated activity (Rotolo and Wilson, 2007; Villar et al., 2013). Women volunteer at higher rates than men (Villar et al., 2013).		

**Qualitative findings**	**Quantitative findings**	**Meta-inferences**
The nature of the activities that, women, and men did when they volunteered was different: women helped organizing events and care for people, and men taught the skills they had learned in their lifelong leisure pursuits. “The senior center keeps me busy because I take people to the doctor. I help people who are older than me. I am a very cheerful person. We do lots of outings, and I am the first one to make them sing” (Blanca, woman) “On Tuesday and Thursday afternoons we practice with the choir. I have been singing for a lone time. We practice an hour a day, though I go an hour earlier to teach others” (Luis, man)	There was a significant difference in the rate women and men volunteered. More than twice the amount of women volunteered than men, with 15% of women volunteering as opposed to 6.2% of men (Pearson Chi- Square = 15.721, *p*-value = 0.000).	The quantitative data converges with the literature (Villar et al., 2013), indicating that women volunteer at rates that more than double of men’s. The qualitative data converges with the literature (Rotolo and Wilson, 2007; Villar et al., 2013), showing that though women volunteer at higher rates, their often take supportive roles, while men take leadership roles. The qualitative data complement the quantitative findings by illuminating the gender segregated nature of the volunteering activities older adults engage in and by pointing at the role of the ethic of care in this segregation.

## Discussion

### Developmental Tasks

We started our analysis with the hypothesis that widowhood and retirement are life events that create discontinuities in behavior (Lopata, 1973; Antonovsky and Sagy, 1990), in this case leisure behavior, and that they influence women and men differently. The qualitative data showed that women reconsidered their lives and made changes in their leisure practices after retirement and, especially, widowhood. These women talked about periods of personal transformation, liberation, and discovery that led them to explore fulfilling new leisure activities. Retirement also played this role in men’s leisure, but to a lesser extent, mostly by allowing them more time to practice leisure activities that they had practiced throughout their adult lives.

The team expected that widowhood would have a larger influence on women’s leisure after later life transitions than on men. This was due to our qualitative findings around the incidence of the ethic of care among women and our knowledge of the culture of the Basque Country and of Spain in general, especially the fact that they grew up and lived in a markedly patriarchal society. However, the quantitative data showed that the differences we expected were not statistically significant in this sample. We speculate that the reasons for this may be attributable to changing gender roles, which are increasingly less differentiated, as some literature has already suggested (e.g., Mailey et al., 2014). We also speculate that the differences by gender in the qualitative findings are due to the particular composition of the women interviewee sample, with half of the group having had traditional gender roles, such as being housewives or having had traditionally feminine jobs, such as seamstress and home care aide. Perhaps a larger sample or samples from other areas of Spain or elsewhere would yield such differences, but the quantitative findings support the conclusion of no significant gender differences in this sample. Clearly more research is needed with other samples, but this mixed method study leads us to have confidence in our conclusions for this group of people.

### Innovation in Leisure

Based on the work by Iso-Ahola et al. (1994) and Nimrod and Kleiber (2007) we suspected, that older women in this study would be more innovative in leisure than men, meaning that they start new leisure activities after later life events more often than men do. As we have seen, women in our study reported that they started new leisure activities more often than men after life events, while men tended to continue the practice of lifelong leisure activities more often than women. Our findings supported this by showing that women had participated in their meaningful leisure activities for shorter periods of time than men. This indicates that meaningful leisure activities of women in this study were newer than those of men, the latter having practiced theirs for some time before these events. These findings indicate that women’s leisure itineraries were characterized by discontinuity more often than in men’s cases. This may be related to the influence of the ethic of care in women’s decisions about their meaningful leisure; fulfilling their tasks associated with gender roles limited their participation and lead to the abandonment of leisure activities for women in this study.

The findings from this study are mostly consistent with previous research (Iso-Ahola et al., 1994; Nimrod and Kleiber, 2007) about continuity and change in leisure later in life: men tended to continue activities they enjoyed in the past or returned to old activities they had abandoned. The women, on the other hand, were more likely to use retirement and, especially, the loss of a spouse as triggers for change (Nimrod and Kleiber, 2007), even though their choices of leisure activities continued to be defined and somewhat constrained by an ethic of care and long-standing domestic responsibilities.

Women’s higher tendency toward innovation seems to be an adaptive mechanism to respond to the discontinuity that characterized their leisure in previous life stages. Given that, among other barriers, the ethic of care prevented them from developing a continued practice of meaningful leisure, they were used to abandoning activities. Consequently, they were more predisposed to initiating new leisure activities after retirement or widowhood. Thus, it seems that women’s orientation to innovation is partly due to the limiting effects of the ethic of care on their leisure in previous life stages.

This would also help explain the differences by gender in the types of leisure innovation we encountered. As we saw in Jaumot-Pascual et al. (2016), women’s innovation in leisure tended to be self-reinvention innovation, where, due to the their discontinuous leisure itineraries, they started new leisure activities that were unrelated to previous ones. On the other hand, men tended to engage in self-preservation innovation, where they started new leisure activities that were related to previous activities. Due to their continuous leisure itineraries, these new activities provided them with opportunities for novelty at the same time they provided certain coherence and continuity with previous activities.

### Ethic of Care as a Constraint to Leisure

Leisure studies literature describes the ethic of care as a constraining factor on women’s leisure involvement by limiting their ability to participate in leisure activities (Henderson and Allen, 1991). We found that while women reported that a broad range of family responsibilities limited their participation in leisure activities, the influence of these responsibilities was much more limited in the case of men. Our findings support Belenky et al. (1986) description of women’s development as relational. Our findings suggest that the ethic of care had a differential role in the abandonment of meaningful leisure activities for women and for men. Both the qualitative and the quantitative findings suggest that an ethic of care had an influence on older women’s leisure and in their abandonment of meaningful leisure activities.

We found that the events that influenced women’s leisure practices go beyond widowhood and immediate family care responsibilities; they included other relational events that broadened both the radius and the type of relationships that are significant enough to have an influence on women’s leisure, such as adult children’s emancipation, separation or divorce, partner’s retirement, and family crises. Men in this study did not mention any of these more extended relational events as having an influence on their leisure.

In general terms, and in light of the results, neither retirement nor widowhood could be characterized in this sample as precipitating a new life stage free of the ethic of care. The ethic of care continues having a strong influence on women’s lives and on their leisure involvement after later life events. It is the liberation from traditionally gendered tasks, and not strictly being retired or widowed, that allowed these women the opportunity for enjoyment and personal development.

### Gender Segregation in Volunteering

We started our integration of findings with two hypotheses: that women volunteer at higher rates than men (Villar et al., 2013) and that volunteering is a gender-segregated activity (Rotolo and Wilson, 2007; Villar et al., 2013). Our qualitative and quantitative findings complemented each other by providing further insights about the role of gender in volunteering among older adults, both in terms of rates of participation and in the content of that participation.

This study shows that women volunteer at a rate that more than doubles that of men and that, although women volunteer at higher rates, they often take supportive roles, often involving the caring for others, while men take leadership roles. This suggests an intimate connection between volunteering and the ethic of care that motivates women to continue prioritizing the needs of others, even in activities that are voluntary. Another way to explain the lower rates of leadership in women’s volunteering involves women’s meaningful leisure discontinuity throughout life. In other words, the need to abandon their meaningful leisure activities due to tasks related to the ethic of care may have prevented the development of skills that would prepare them to become leaders or to transfer knowledge, which are more common among men.

Unfortunately, the questionnaire did not include an item on the content of participants’ volunteering activities, which prevented our exploring gender differences in the content of volunteering activities. This would be an interesting direction to explore in future research.

## Conclusion

In this secondary analysis, using mixed methods research through joint displays, qualitative data have provided contrast, complement, and depth to some of the results we obtained in the quantitative analysis. This has confirmed the differential influence of widowhood and retirement on older adults’ leisure according to gender. Although we cannot conclude that widowhood has a larger influence than retirement on women, we can state that the ethic of care continues being a constraint that limits women’s meaningful leisure opportunities in later life. For women, neither retirement nor widowhood meant the beginning of a new vital stage that is untethered by the ethic of care. It is the liberation from the tasks associated to gender roles, and not strictly being a retired or widowed that allowed them to find an opportunity for enjoyment and personal development in leisure.

The results also show that in order to understand the influence of the ethic of care on the leisure of older adults, and particularly women, we need to take into account their leisure in previous stages in life. The need to fulfill traditionally gendered tasks throughout life for women more than for men has meant the abandonment of meaningful leisure activities. This lack of continuity for women’s leisure contrasts with the continuity of men’s. This has prevented the development of leisure-related skills, which often influences their ability to develop leadership roles. However, this discontinuity also predisposes them to start new leisure activities and, thus, the need for innovation in later life. Finally, gender and the ethic of care also influence volunteering. On the one hand, it influences levels of participation by gender, with women participating more than men. And on the other hand, it influences the content of those activities, which are highly gendered and directed toward providing care and serving the community.

The expectation is that the influence of gender on leisure choices will diminish in the next generations, as the literature is starting to show. However, its influence is a continued reality for the current generation of older adults in the Basque Country. The challenge resides in providing the conditions for gender to stop being a variable that perpetuates inequality in the context of leisure.

## Author Contributions

NJ-P and MJM conceived the aim of the paper and included the gender perspective from which the results have been interpreted. NJ-P took the lead in the mixed method design. JC contributed to the data collection and the analysis of the results. DAK was involved in the designing, planning, and supervision of the work, helped specially to develop the theoretical framework, and gave support during all the phases of the data analysis and writing of this paper. NJ-P and MJM were responsible for drafting the work and revising it critically for important intellectual content, but all authors discussed the results and contributed to the final manuscript.

## Conflict of Interest Statement

The authors declare that the research was conducted in the absence of any commercial or financial relationships that could be construed as a potential conflict of interest.
